# Stability of Iridium Single Atoms on Fe_3_O_4_(001) in the mbar Pressure Range

**DOI:** 10.1021/acs.jpcc.3c03097

**Published:** 2023-09-14

**Authors:** Nicolo Comini, J. Trey Diulus, Gareth S. Parkinson, Jürg Osterwalder, Zbynek Novotny

**Affiliations:** † Physik-Institut, Universität Zürich, Zürich CH-8057, Switzerland; ‡ Swiss Light Source, Paul Scherrer Institut, Villigen-PSI CH-5232, Switzerland; § Institute of Applied Physics, TU Wien, Vienna A-1040, Austria; ∥ EMPA, Laboratory for Joining Technologies and Corrosion, Swiss Federal Laboratories for Materials, Dübendorf CH-8600, Switzerland

## Abstract

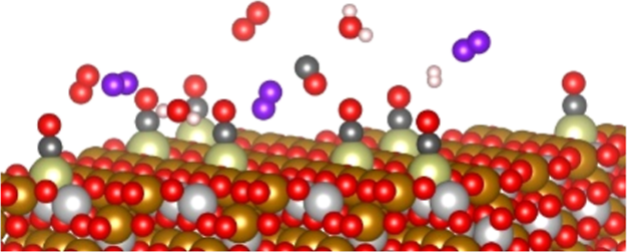

Stable single metal adatoms on oxide surfaces are of great interest for future applications in the field of catalysis. We studied iridium single atoms (Ir_1_) supported on a Fe_3_O_4_(001) single crystal, a model system previously only studied in ultra-high vacuum, to explore their behavior upon exposure to several gases in the millibar range (up to 20 mbar) utilizing ambient-pressure X-ray photoelectron spectroscopy. The Ir_1_ single adatoms appear stable upon exposure to a variety of common gases at room temperature, including oxygen (O_2_), hydrogen (H_2_), nitrogen (N_2_), carbon monoxide (CO), argon (Ar), and water vapor. Changes in the Ir 4f binding energy suggest that Ir_1_ interacts not only with adsorbed and dissociated molecules but also with water/OH groups and adventitious carbon species deposited inevitably under these pressure conditions. At higher temperatures (473 K), iridium adatom encapsulation takes place in an oxidizing environment (a partial O_2_ pressure of 0.1 mbar). We attribute this phenomenon to magnetite growth caused by the enhanced diffusion of iron cations near the surface. These findings provide an initial understanding of the behavior of single atoms on metal oxides outside the UHV regime.

## 1 Introduction

Current advancements in the field of catalysis have focused on two main research topics, specifically highly selective functionalized molecules and nanostructured materials. The former topic is individually geared toward specific reactions, selecting well-defined active centers, such as specific catalytically active atoms and the sites in their vicinity, where the catalytic mechanism can be fully understood.^1^ Nanostructured surfaces sacrifice this active atom specificity in favor of a wider range of applications with the aim to enhance the effectiveness and selectivity of the catalyst.^2^ This is particularly important for precious metals, where limited availability and high costs impede their widespread use.

Research on nanostructured materials aims to improve catalyst technology by enhancing the interactions between the catalyst and the reactant molecules. A well-known factor in the interaction between reactant and catalyst is the latter’s particle size; stronger adsorption is often found on smaller nanoparticles^3^ due to a strong metal–support interaction between the nanoparticle and the oxide support.^4^ In the ultimate limit, isolated adatoms stabilized on a support, a “so-called” single-atom catalyst (SAC), minimize the amount of material required. Moreover, if the individual atoms are dispersed on the supporting surface with the same unique coordination, they may exhibit higher selectivity than particles exposing various coordinations.^5^ Thus, SACs can combine the efficiency of heterogeneous catalysis with the selectivity of homogeneous catalysis if all single-atom sites are identical.^6,7^ Reactions taking place on these SACs will still be influenced by the support, as their anchoring site and sites in their vicinity can play a role in terms of site accessibility or adsorption. The adsorption site geometry may favor or hinder the ideal reactant binding to the active site, while defining a single catalytic pathway if the only active sites belong to the metal adatom. As an electronic effect, charge transfer between metal atom and support affects the oxidation state of the catalyst and the confined electrons will display a specific distribution of energy levels.^5^

The (001)-oriented surface of magnetite, Fe_3_O_4_, has been identified as one of the most promising substrates to stabilize SACs on metal oxide surfaces.^8^ Ordered subsurface cation vacancies and interstitials cause a (√2 × √2)R45° reconstruction,^9^ resulting in a surface exhibiting undulating rows of Fe and O atoms. The reconstruction creates distinct adsorption sites that stabilize single metal atoms, as initially discovered in the case of gold,^10^ and later followed by many other metals.^8,11,12^ Single metal atoms adsorbed on a reconstructed Fe_3_O_4_(001) surface show a high degree of stability against thermally induced sintering. For example, the onset of sintering has been observed for Au adatoms when exposed to temperatures above 700 K^8,10^ where the (√2 × √2)R45° reconstruction is progressively lifted, up to 788 K where the reconstruction disappears completely.^13^ For Pt and Pd adatoms, carbon monoxide-induced sintering has been reported at 300 K.^8,11^ Pd adatoms were reported to dissociate H_2_ heterolytically between Pd adatom and surface oxygen.^14^ Pd adatoms also lower the barrier to C–H bond cleavage by a factor of 2 compared to a clean Fe_3_O_4_(001) surface, as was demonstrated by low-temperature oxidation of methanol to formaldehyde.^15^ Finally, Rh adatoms were reported to allow for CO oxidation via the Mars–van Krevelen mechanism.^16^ The single-atom catalysis on substrates other than Fe_3_O_4_(001) is further described in numerous review articles.^5,17^

The properties of iridium,^18^ specifically its limited availability and important role in catalysis,^19−23^ suggest it being a good candidate for SACs. Recently, catalytic oxidation of methane (CH_4_) over low-coordination Ir sites on both IrO_2_(110) and Ir(001) was reported by a combined ambient-pressure X-ray photoelectron spectroscopy (APXPS) and density functional theory (DFT) study.^24^ While CH_4_ activation is facile on the metallic Ir(100) surface, it can get readily poisoned by adsorbed oxygen since O_2_ dissociation is even more facile, which is not the case for the IrO_2_(110) surface.^25^ IrO_2_ nanoparticles larger than 1 nm on an alumina support are an excellent catalyst for selective catalytic oxidation of ammonia with a 99% efficiency,^26^ while Ir_1_ single atoms can greatly enhance the reducibility of the FeO_*x*_ support and generate oxygen vacancies, leading to the excellent performance of the Ir_1_/FeO_*x*_ single-atom catalyst for water–gas shift reaction.^27^

The Ir_1_–Fe_3_O_4_(001) model catalyst was studied previously under ultra-high vacuum (UHV) by means of scanning tunneling microscopy, X-ray photoelectron spectroscopy (XPS), and DFT.^12^ In this work, we investigate iridium single atoms (Ir_1_) on a Fe_3_O_4_(001) surface by exposure to O_2_, CO, H_2_O, H_2_, N_2_, and Ar at partial pressures of up to 20 mbar, extending the existing UHV studies^12,28−30^ toward a more realistic pressure range under moderate temperatures (300–573 K). The Ir_1_ single adatoms appear stable during the gas exposure, although the changes in the Ir 4f binding energy suggest that the adatoms interact not only with adsorbed or dissociated molecules but also with water/OH groups and adventitious carbon species displaced from the chamber walls under these pressure conditions. The Ir_1_ adatoms are stable unless exposed to oxidizing conditions (at least 0.1 mbar O_2_ at 473 K), where Ir_1_ adatom encapsulation takes place by magnetite growth caused by the enhanced diffusion of iron cations near the surface.

### 1.1 Background

At room temperature, Ir_1_ is stable in a two-fold configuration bound to surface oxygen atoms. Upon mild annealing to 623 K, Ir_1_ first migrates into the surface, substituting a five-fold coordinated Fe atom, which in turn occupies an octahedral vacancy in the subsurface layer.^12^ When the temperature increases to 723 K, Ir migrates into the subsurface region, setting a temperature limit for this system when used as a SAC. When CO is dosed in UHV, it adsorbs to the Ir_1_ adatoms and strengthens its interaction with the surface, preventing the adatom’s incorporation into the surface until desorbing the CO molecule (or oxidizing it to CO_2_) at ∼600 K.^12^ On the other hand, CO interacts weakly with the pristine Fe_3_O_4_(001) surface, with the strongest adsorption occurring at surface defects and desorption taking place well below the room temperature, with no sign of surface reduction or carburization.^30^

The interaction of a bare Fe_3_O_4_(001) surface with CO, H_2_O, O_2,_ H_2_, and N_2_ has also been studied previously.^8,31−34^ Oxygen dosing has mostly been studied from the perspective of magnetite oxidation. Annealing of Fe_3_O_4_ to 573 K in air produces a capping layer of α-Fe_2_O_3_, with the (001) surface oxidizing faster than (111) or (110).^35^ In the presence of active centers for O_2_ dissociation, such as Pt clusters, the resulting O atoms were observed being able to move to the support and react with excess Fe atoms diffusing from the bulk to the surface, forming new Fe_3_O_4_(001) islands.^29^ Water instead initially dissociates into surface hydroxyls when adsorbing at an oxygen vacancy site on the Fe_3_O_4_(001) surface. Cooperative interactions among water molecules further adsorption in less favorable sites, reaching a saturation coverage at a pressure of ∼10^–2^ mbar, which may then progress into molecular water adsorption.^34^ At low temperatures, it is also possible to form ordered structures on both the (001) and (111) magnetite surface before forming an amorphous solid water layer.^36^ In UHV, water desorbs from the Fe_3_O_4_(001) surface below 250 K,^37^ but above 10^–5^ mbar the subsurface cation vacancy reconstruction has been reported to be lifted and to revert to a (1 × 1) structure, whereupon an ordered oxyhydroxide phase grows that passivates the surface and prevents further water dissociation.^31^ Molecular hydrogen (H_2_) does not adsorb onto or reduce the (001) surface at 300 K but can adsorb forming surface hydroxyl groups, if cracked thermally using a hot tungsten filament.^8,32^ N_2_ dissociative adsorption was found to occur on the (001) surface on octahedral Fe^3+^ and on top of surface O atoms already below 10^–5^ mbar, increasing with gas pressure.^33^ A link to the number of defect sites was proposed, with the active sites assigned to Fe^3+^ being reduced to Fe^2+^, as identified from the valence band spectra.

## 2 Experimental Details

### 2.1 XPS Analysis

Experiments were conducted at the in situ spectroscopy beamline of the Swiss Light Source (SLS), using the solid–liquid interface chamber endstation.^38^ XPS spectra were acquired in a non-baked chamber (base pressure below 1 × 10^–8^ mbar, termed HV in the following section), using a linearly polarized beam with a photon energy of 950 eV, a Scienta R4000 HiPP-2 analyzer with an entrance cone aperture diameter of 500 μm, and a working distance of 1 mm, operated in an angular lens mode using a 50 eV pass energy. Sample heating during XPS experiments was performed utilizing a pyrolytic boron nitride insulating ceramic and pyrolytic graphite as a resistive heating element, with the temperature determined using a Pt100 sensor.^38^ The binding energy (BE) was calibrated with reference to the Fe_3_O_4_ substrate using the O 1s peak. Such an approach was adapted due to occasional fluctuations of the photon energy (timescale of hours) and allowed us to maintain consistent results with Au 4f-based energy calibration for a stable beam. For polycrystalline polished gold, the Au 4f_7/2_ peak position was set at 84.0 eV with a full-width at half maximum (FWHM) of 1.34 eV under our experimental conditions.

### 2.2 Sample Preparation

Fe_3_O_4_(001) natural single crystals (SurfaceNet GmbH) were prepared in a separate UHV chamber with a base pressure of 2 × 10^–10^ mbar. Sample preparation consisted of cycles of Ar^+^ sputtering (a mean kinetic energy of 950 eV, 30 min) and annealing performed in alternation in 5 × 10^–7^ mbar of molecular O_2_ for 10 min, and UHV annealing at 1100 K for 15 min. The (√2 × √2)R45° structure was verified by low-energy electron diffraction (LEED) using an electron beam current of nominally 40 nA, and detection multiplied by a pair of multichannel plates (MCPs) with an 800 V potential applied. The image shown in Figure S1 has been processed using a Spherize plugin in Adobe Photoshop to remove the distortion induced by the nonspherical field caused by the planar MCPs. Iridium was deposited on a sample kept at room temperature (below 310 K) from a 2 mm thick Ir rod (99.99%) using an electron beam evaporator (EFM3, Focus GmbH) calibrated with a water-cooled quartz crystal microbalance (Inficon) placed at the same position as the sample during evaporation. For consistency with earlier work,^10^ we define the monolayer (1 ML) coverage of Ir_1_ as one iridium atom per surface unit cell (UC, 8.4 × 8.4 Å^2^). Unless stated otherwise, the Ir_1_ coverage is approximately 0.6 Ir_1_ atoms/UC. Given the reported instability of Ir dimers on the (001) surface^12^ and the amount of Ir deposited, predominantly single Ir_1_ adatoms (<80%) should be present on the surface.

### 2.3 Gases and Liquids

MilliQ water (Type 3) was purified by four freeze–pump–thaw cycles using liquid nitrogen for freezing and a turbomolecular pump for pumping. The water was contained in a glass vial permanently attached to the analysis chamber via a high-precision leak valve, which allowed the experimental chamber to be backfilled with up to 20 mbar of gas phase water.^39^ O_2_, Ar, and H_2_ were dosed through a similar high-precision leak valve from a Minican bottle (PanGas, 5N), at pressures ranging from 1 × 10^–5^ mbar to 1 mbar. H_2_ dosing was performed with the gas reservoir in contact with a cold trap kept at liquid nitrogen temperature. N_2_ was dosed from a gas cylinder (PanGas, 6N) through a separate high-precision leak valve. For CO, a Minican (PanGas, 5N) was attached to a separate dedicated dosing system attached to the experimental chamber using an all-metal valve (VAT Vatring). The CO gas leak rate was controlled via a flow meter, followed by a heated (600 K) copper trap to avoid possible Ni(CO)_4_ contamination.

### 2.4 Data Analysis

Spectra integration and plotting were done using Igor Pro 6.37. Peak fitting was performed in CasaXPS V.2.3.19 and Igor Pro 6.37. Cross sections and asymmetry parameters were obtained by linearly interpolating the values tabled by Lindau and Yeh,^40^ utilizing the differential cross sections for horizontal, linearly polarized light described by eq 9 in ref (41). To determine the iridium coverage, an ultrathin film approximation model is adopted.^42,43^ The coverage *N*_Ir_ of iridium, in number of adatoms per surface unit cell, is referenced to the Fe_3_O_4_ surface unit cell by eq 1
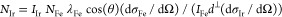
1here, *I*_x_ represents the peak intensity, λ_x_ is the inelastic mean-free path (IMFP) of electrons in the Fe_3_O_4_(001) substrate determined by the TPP-2M model,^44^ dσ_x_/dΩ is the differential cross section, θ is the polar emission angle, and *d*^⊥^ is the interplanar distance for the Fe_3_O_4_(001) lattice. The Ir 4f_7/2_ and Fe 3p core levels were selected for a coverage analysis since both can be measured simultaneously in a narrow energy window. Consequently, attenuation through an adventitious carbon layer or through the gas phase will not change the intensity ratio because the kinetic energies are similar. The IMFP for Fe 3p photoelectrons is 18.25 Å, which is larger than the distance between adjacent octahedral layers. Therefore, we chose not to evaluate the Fe 3p photoemission intensity layer-by-layer but averaged the number N_Fe_ of Fe atoms per layer to 6, with a layer distance d^⊥^ of 2.1 Å.

## 3 Results and Discussion

### 3.1 Benchmark Spectra under HV Conditions

**Figure 1:**
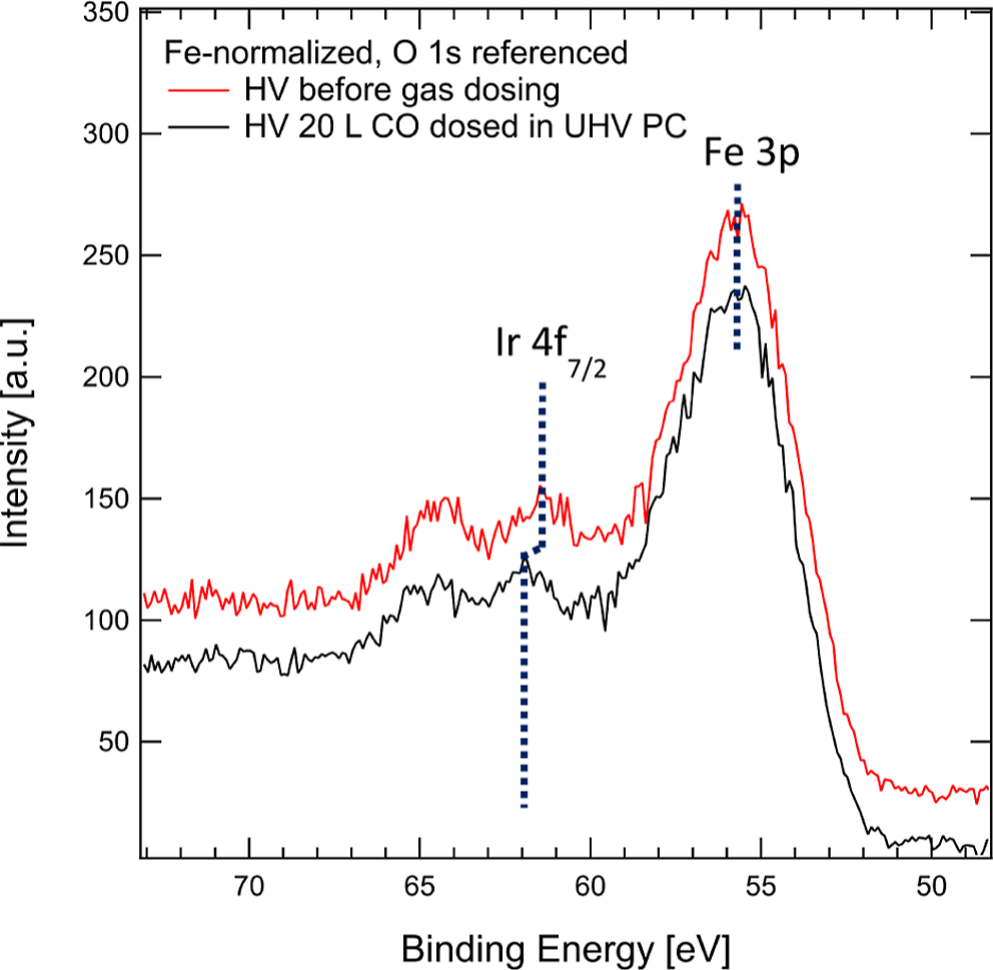
XPS spectrum from an as-prepared Fe_3_O_4_(001) sample with 0.6 Ir_1_ adatoms/UC (red) and a similarly prepared sample additionally exposed to 20 L of CO (black). The Fe 3p peak is characterized by a highly asymmetric shape, partially overlapping with the Ir 4f doublet. A vertical offset is applied to avoid spectra overlap.

Before investigating Ir_1_ adatoms under near-ambient pressure conditions, we repeated the experiments previously performed by Jakub et al.^12^ using synchrotron-based XPS. Our goal was to obtain reference spectra for two-fold coordinated Ir_1_–Fe_3_O_4_(001), since this is a starting point for all subsequent experiments. Figure 1 shows the XPS spectrum covering the Fe 3p and Ir 4f regions acquired in HV for two different sample preparations: as-prepared clean surface following deposition of 0.6 Ir_1_ atoms/UC, and another, freshly prepared sample with 0.6 Ir_1_/UC, additionally exposed to 20 L of CO in the UHV preparation chamber.

The Ir 4f doublet shows the 4f_7/2_ peak centered at 61.5 eV for the UHV preparation, while after CO exposure, the peak is observed at 61.9 eV. Jakub et al.^12^ reported the 4f_7/2_ peak BE of Ir_1_ on the UHV-prepared surface at 61.1 and 61.5 eV for the CO exposed sample where a single CO molecule is attached to every Ir_1_ adatom. A higher BE (62.1 eV) was reported for five-fold coordinated Ir atoms incorporated within the Fe_3_O_4_(001) surface,^12^ but such in-surface incorporation requires a thermal annealing to 500 K, whereas we deposited Ir_1_ at room temperature. While we observe a similar +0.4 ± 0.1 eV BE shift following CO dosing, our Ir 4f BEs are offset by an additional +0.4 ± 0.1 eV toward higher BE compared to ref (12). To understand the origin of this BE offset, we want to emphasize that our experiments contain a much higher amount of adventitious carbon (see Figure S2 and the adventitious carbon quantification shown in Figure S5) compared to ref (12), which is unavoidable under our experimental conditions.^39^

Considering the Ir–C bond in the adsorption geometry of CO on Ir_1_,^12^ we propose the following explanation for the shift of our Ir 4f peaks toward higher BE. Ir_1_ adsorbed in a two-fold configuration on the (√2 × √2)R45°-reconstructed Fe_3_O_4_(001) surface is characterized by a BE of 61.1 eV.^12^ Residual gas present in the HV environment of the experimental chamber may hydroxylate the surface, which has been reported to enhance the surface’s reactivity with carbon species.^45^ An adventitious carbon atom binding to the Ir_1_, or adsorbing on the sites in its vicinity, increases the Ir_1_ coordination and leads to a change in the local potential, similar to the carbonyl bond of a CO molecule. A CO molecule may still remain adsorbed on Ir_1_, as steric and electrostatic repulsion between C and CO is much less than that between two CO molecules. This could then result in a Ir_1_–OC_2_ complex, which leads to 5-fold coordination of iridium due to the additional bond between Ir_1_ and the underlying subsurface O following CO adsorption.^12^ A similar coordination, such as five-fold coordinated Ir atom incorporated within the Fe_3_O_4_(001) surface (where it substitutes octahedrally coordinated Fe), was determined by DFT to be more stable than two-fold Ir_1_ by approximately 1 eV (see energy diagram in Figure S2 in ref (12), which compares the stability of Ir_1_ adatom, in-surface five-fold Ir, and six-fold Ir in the second and third octahedral layers, for more details). The increased coordination number of Ir_1_ leads to a higher Ir 4f BE upon adventitious carbon and water/OH uptake, as shown in later sections in this manuscript, compared to the same Ir_1_ single atoms on a perfectly clean Fe_3_O_4_(001) surface. While this explanation of the BE shift is the most straightforward, future imaging studies will be needed to rule out other possible alternatives, such as adsorption of the oxidizing species from the background gas, clusters involving Ir and C with lower coordination to the substrate, or carbon deposition changing the local electronic structure of the Fe_3_O_4_(001) substrate to name a few.

### 3.2 Exposure to CO, O_2_, and Ar

**Figure 2:**
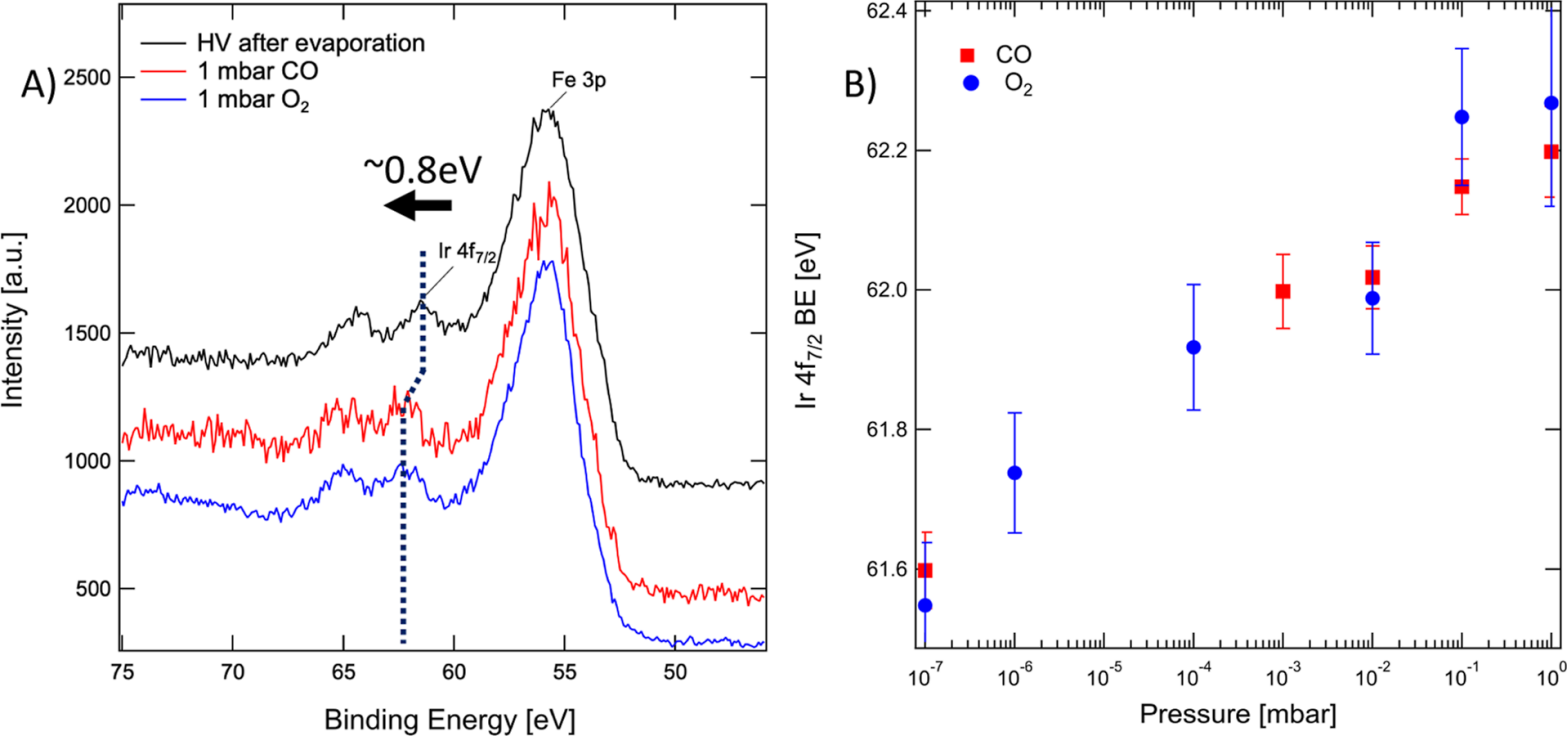
(A) Substrate-normalized XPS spectra of the Fe 3p/Ir 4f region for a sample as-prepared (black) and during exposure to 1 mbar CO (red) or 1 mbar O_2_ (blue), where the Ir 4f doublet is shifted toward higher BE. (B) BE of the Ir 4f_7/2_ peak plotted as a function of pressure.

The Ir_1_–Fe_3_O_4_(001) system was subsequently exposed to a variety of gases, to approach realistic conditions for catalyst use. Aside from increased adventitious carbon contamination,^39^ we observe a shift in the Ir 4f BE following the increase in pressure. We report in Figure 2 this observation for CO and O_2_. A similar effect takes place for exposure to all probed gases at 1 mbar (CO, O_2_, N_2_, Ar, H_2_O, and H_2_), although with varying magnitudes. The FWHM of the Ir 4f peak does not change significantly, which indicates that we do not see a superposition of two peaks changing in relative intensity. Interestingly, such a shift toward higher BE is observed even in the case of an inert gas (1 mbar of Ar), although to a smaller extent (61.9 ± 0.1 eV in 1 mbar Ar, against 62.2 ± 0.1 eV in 1 mbar CO). A small increase in the higher BE shoulder of the O 1s peak can be observed following Ar exposure (see Figure S3), aside from adventitious carbon buildup.

Identifying the precise cause of this BE shift is not trivial, since we observed a different magnitude of the BE shift for each exposed gas. While a plausible explanation would be the different nature of interaction of probed molecules with Ir_1_ and the Fe_3_O_4_(001) substrate, the observed shift of Ir 4f toward higher BE during exposure to 1 mbar of Ar rules out the interaction between the gas molecules and the substrate as the sole factor responsible for the observed Ir 4f BE shift. At the same time, due to the low Ir_1_ coverage, it is not possible to clearly separate spectral contributions from species adsorbed on Ir_1_ or on the rest of the substrate. The O 1s and C 1s have an identical peak envelope regardless of Ir_1_ presence (see Figure S4) due to the low adatom coverage and relative photoionization cross sections. As such, within our experimental limits, we cannot univocally attribute this shift to a change in electrical potential specific to adsorbed molecular species from the probed gasses. The gradual shift of the Ir 4f BE with pressure appears to be due to a combination of molecule adsorption and adventitious carbon contamination. The correlation between the Ir 4f BE and the carbon coverage is reported in Figure S5. When exposing Ir_1_ to 1 mbar of Ar (the last point of the respective data set in Figure S5), a milder shift (compared to CO and O_2_, as shown in Figure 2B) is observed. Argon will not directly adsorb, yet at higher pressures could contribute to the buildup of adventitious carbon^39^ due to impurities in the 10 ppm range contained in the Ar gas or added to it during the dosing (see Figure S5; raw data including the C1s region can be accessed using the link in the Data Availability section). By comparing the carbon coverage and the Ir 4f BE shift, we observe approximately +0.15 eV shift per 1 Å of carbon deposited. The curves for O_2_ and CO in Figure 2B lead to a greater total peak shift which suggests that for these gases a possible adsorption of molecules on the Ir_1_ adatoms could additionally contribute to the observed Ir 4f BE shift, although the precise identification of the exact role of such additional adsorbates and their adsorption sites are not possible. The high BE shoulder observed in the O 1s spectra shown in Figure S4a correlates with the corresponding 288.5 eV BE peak in the C1s spectrum (shown in Figure S4b) in a 2:1 ratio, suggesting their identification as COO^–^ groups. Such carbon species present up to a coverage of approximately 5 COO^–^ groups/UC are not present after Ar dosing, nor can such species be resolved after 20 L CO dose in UHV. On the UHV CO-dosed sample, the coverage of COO^–^ groups increases to approximately 1 COO^–^ group/UC after backfilling the chamber to 1 mbar O_2_, suggesting an adventitious origin for such adsorbates.

A special mention can be made regarding the exposure to a high pressure of CO. During CO exposure, the Ir 4f BE (62.0 ± 0.1 eV) measured at 1 × 10^–3^ mbar CO (second point of the corresponding data set in Figure S5) is compatible with HV BE measured after the 20 L CO dose (61.9 ± 0.1 eV), where the CO dosing was performed in UHV immediately after sample preparation in the better vacuum of the UHV preparation chamber. This second measurement is represented in Figure S5 by the first point of the data set shown in yellow. This compatibility indicates that, even with some adventitious carbon present on the surface, gas-phase CO is still eventually able to bind to Ir_1_ as indicated by the BE shift of +0.4 ± 0.1 eV. Conversely, adventitious carbon can still coordinate with the Ir_1_ after CO adsorption in UHV. This indicates that much of the adventitious carbon is present in the form of large, three-dimensional clusters. The BE shifts observed at higher pressures are instead consistent with an adventitious carbon-induced change.

This change in Ir 4f BE also remains stable over time in a vacuum. Ir_1_ exposed to 1 mbar CO does not show any detectable change in the binding energy of Ir 4f after being stored for 36 h in UHV (Figure S6). While CO desorption from Ir_1_ is expected to take place only at 600 K,^12^ relaxation or desorption of the carbon layer could take place at lower temperatures. To investigate this possibility, we heated the sample temperature up to 473 K in HV while collecting the XPS spectra. From the results shown in Figure S7, we observe that the Ir 4f BE is slightly reduced as temperature increases, suggesting that some structural rearrangement in the adsorbate layer takes place. No significant change is observed in the C 1s spectrum at these higher temperatures.

### 3.3 Exposure to H_2_

We further studied the Ir_1_–Fe_3_O_4_(001) model system upon exposure to molecular hydrogen at higher pressures. While Parkinson et al.^32^ studied adsorption of atomic H on the surface, to our knowledge, no experiments have been conducted at mbar pressures of H_2_ or in the presence of Ir_1_ on the surface. No chemical change in the Fe 3p or O 1s regions (see Figure S8) can be resolved even after exposure to 1 mbar H_2_ aside from a slight shift of the Ir 4f BE (see Figure S5, where the BE shift is plotted against the carbon thickness), suggesting that no significant surface reduction takes place at room temperature even in the presence of Ir_1_.

### 3.4 Exposure to N_2_

**Figure 3:**
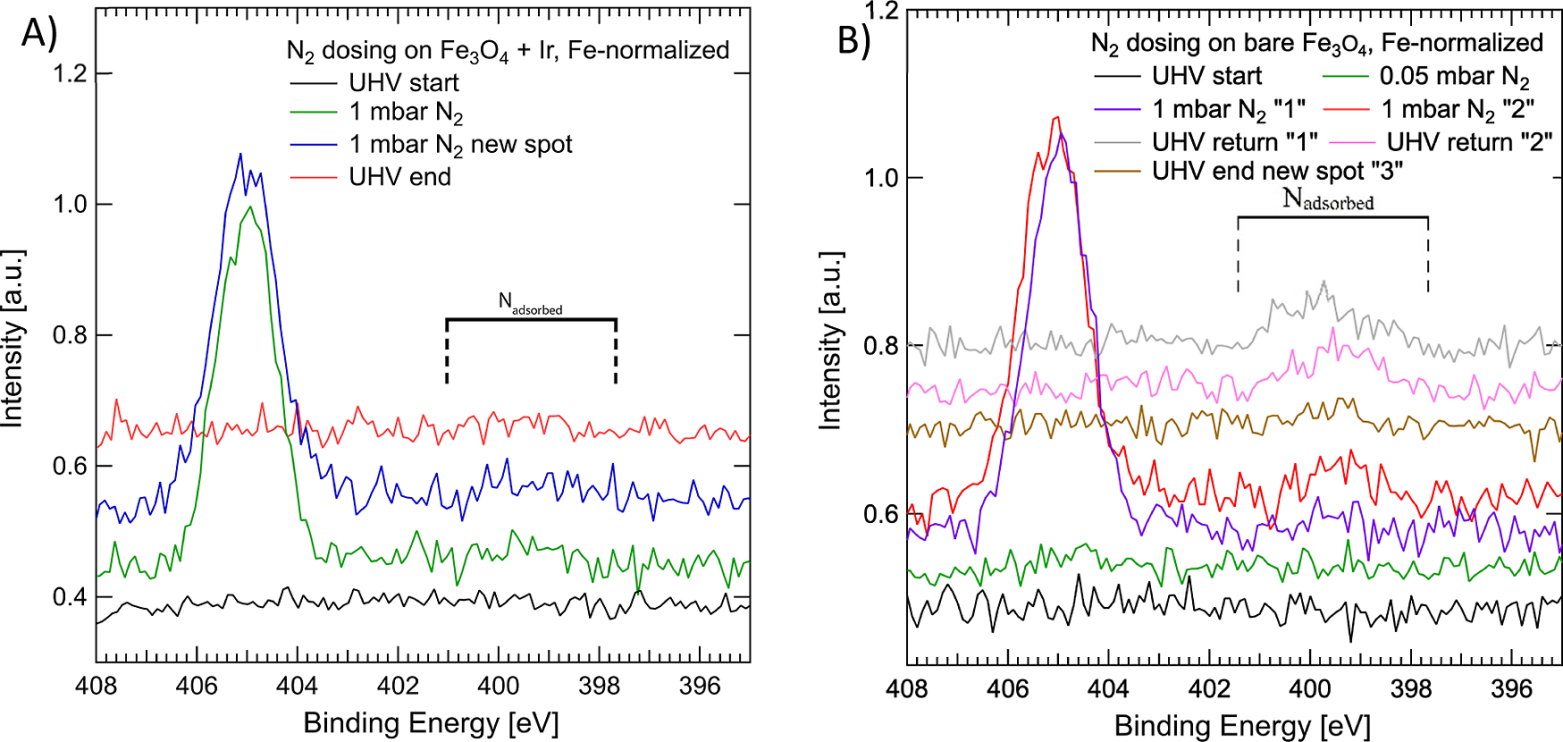
APXPS spectra of the N 1s region for a Ir_1_–Fe_3_O_4_(001) sample (A) and bare magnetite (B). (A) No clear adsorbed N species appear during the first experiment. (B) On bare magnetite, a different emission profile appears after exposure in different locations on the surface. In 1 mbar N_2_ on two spots “1” and “2” at a relative distance larger than 0.5 mm a different line shape envelope is detected, where no N appears in the first spot. A gas-phase N_2_ peak is observed at 405 eV. A different shape appears upon revisiting these spots after evacuating the gas. It is also possible to find an area “3” without adsorbed N 1s emission (brown).

In the Ir_1_–Fe_3_O_4_(001) model system, the role of Ir_1_ in N_2_ adsorption is not known but can be compared to the situation on bare magnetite that has been studied by Degaga et al.^33^ We performed a similar experiment by exposing a bare Fe_3_O_4_(001) surface and the same surface decorated with Ir_1_ to different pressures of N_2_ up to 1 mbar. Figure 3A shows no clear N_2_ adsorption or dissociation at 300 K on the Ir_1_–Fe_3_O_4_ system. A faint signal located at 399 eV, corresponding to less than 1 N atom/UC, can be detected in 1 mbar N_2_, and none was observed at lower pressures or upon returning to HV.

Figure 3B shows our reproduction of the N_2_ exposure experiment by Degaga et al.^33^ on a bare Fe_3_O_4_(001) substrate (without Ir_1_). Interestingly, we observe different intensities and peak shapes of N 1s emission at different spots on the sample surface. This experiment indicates a dependency on the specific local condition of the surface, the extent of N_2_ dissociation, and the type of bonds N makes with the surface. Looking at the C 1s emission, we can also conclude that, within the experiment, the amount of adsorbed N does not seem to be correlated with the amount of carbon on the surface (Figure S9). We propose that the local density of surface defects, such as anti-phase domain boundaries,^46^ step edges,^47^ areas containing a Fe-rich surface,^48^ or a different amount of adventitious carbon contamination, may be responsible for N_2_ dissociation and explain the possible difference between our data and the in situ/ex situ experiments reported in ref (33). In their study, Degaga et al. during sample preparation used a higher energy of Ar^+^ ions during sputtering (1.5 keV with Ar^+^ ion current of 11 μA against 950 eV and 1 μA used in this work, although for spectroscopic experiments, a similar Ar^+^ ion energy was used), which may lead to a Fe-rich termination of the (001) surface.^49^ In addition, it is not mentioned in the work^33^ whether after O_2_ annealing, the sample was cooled in UHV or in a partial pressure of oxygen. The latter case can lead to a higher density of islands on the surface and, consequently, more step edges,^47^ which could explain the discrepancy between our results and ref (33). Our data do not provide any evidence for enhanced reactivity with N_2_ on the Fe_3_O_4_(001) surface when decorated with two-fold coordinated Ir_1_ adatoms. We observe rather the opposite effect that with Ir_1_, we cannot detect any nitrogen on the surface. Since the active sites for N_2_ dissociation were identified to be octahedrally coordinated Fe^3+^ in ref (33), the presence of positively charged^28^ Ir_1_ results in the reduction of active sites for N_2_ dissociation (since the surface Fe cations are reduced toward Fe^2+^), which is reflected by the absence of the N 1s signal in Figure 3a.

### 3.5 Exposure to Water Vapor

**Figure 4:**
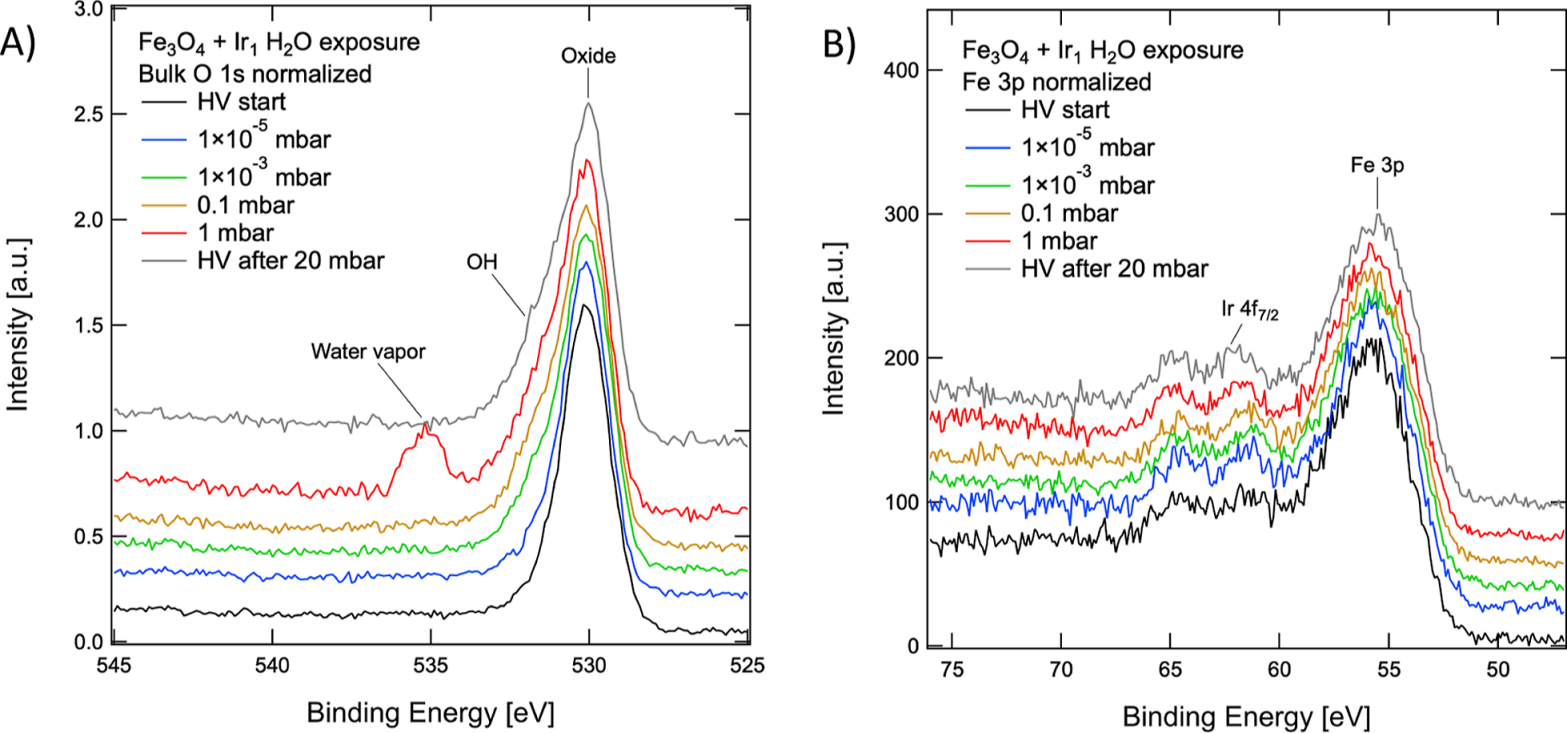
XPS spectra of the O1s region (A) and Fe 3p/Ir 4f region (B) during water vapor exposure experiments, substrate normalized. (A) As water pressure increases, in the high BE side of the O 1s peak, a shoulder associated with surface hydroxyls (OH) and some oxygen-containing carbon species increases. At a pressure of 1 mbar, a strong signal from the gas-phase water appears at 535.0 eV. (B) Ir_1_ adatoms appear stable on the surface upon water exposure, and only the shift in the Ir 4f BE can be observed. The spectra displayed following 20 mbar H_2_O exposure (grey) were acquired after annealing to 473 K.

The Ir_1_–Fe_3_O_4_(001) system has also been exposed to water vapor. Figure 4 shows the Fe 3p/Ir 4f region during vapor exposure in pressure steps up to 1 mbar. In addition, the sample was exposed to approximately 20 mbar of water vapor in equilibrium with liquid water while annealing it to 473 K, with the analyzer’s entrance orifice capped with a Viton seal (since the maximum working pressure for an orifice with a 500 μm diameter is 5 mbar).^38^ The sample was then measured later at room temperature after restoring the HV conditions.

During these experiments, we observe hydroxylation of the surface and adsorption of COOH-like species manifested by an additional peak located at ∼531.5 eV (we note that a similar extent of hydroxylation and contamination was observed when dosing other gases such as CO or O_2_—see Figure S4), but no significant change in the Fe 3p peak or Ir 4f intensity. Ir_1_ thus appears to remain stable in water vapor and bonded to the surface. It should be noted that the experiments involved only gas-phase water, and the surface was not put in contact with liquid water. The difference of interactions with the liquid may be significant,^50^ but the constant Ir 4f signal intensity in XPS suggests that Ir_1_ may also survive in its well-defined adsorption site when exposed to liquid water.

### 3.6 Gas Exposure at Elevated Temperatures

**Figure 5:**
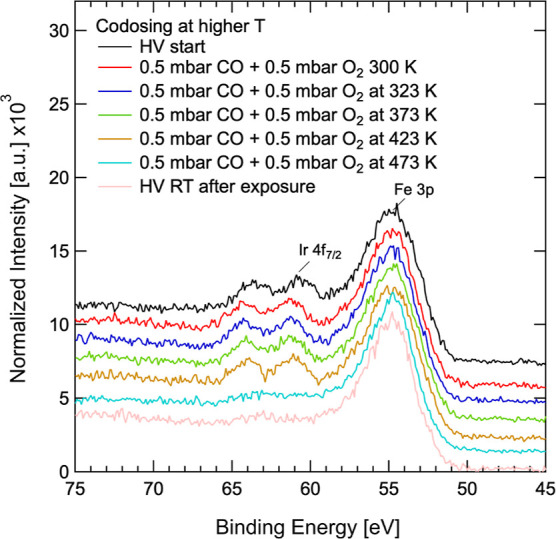
Substrate-normalized XPS spectra of the Fe 3p/Ir 4f region during CO–O_2_ co-exposure at different sample temperatures. The Ir 4f peaks disappear upon reaching 473 K. After removing the gas, returning to room temperature in vacuum and moving to a fresh spot on the surface (distance of 1 mm), still no Ir 4f intensity can be detected.

To complete this investigation, we performed gas-phase exposure experiments at temperatures up to 473 K. The Ir_1_–Fe_3_O_4_(001) model system was exposed to a 50:50 gas mixture of CO and O_2_ of 1 mbar total, then individually exposed to O_2_ and CO up to 1 mbar and H_2_O vapor up to 20 mbar. During CO and O_2_ co-dosing, no reaction products from the oxidation of CO were detected by XPS and quadrupole mass spectrometry (QMS) with the quadrupole probe located between differential pumping stages of the analyzer. While a small quantity of CO_2_ may have been produced, a partial pressure below 0.1 mbar is too low to produce sufficient XPS emission from the gas phase and the sensitivity of the QMS is limited to a similar extent. However, during co-dosing of 0.5 mbar O_2_ and 0.5 mbar CO, we observed a disappearance of the Ir 4f intensity when the temperature reached 473 K, as shown in Figure 5.

**Figure 6:**
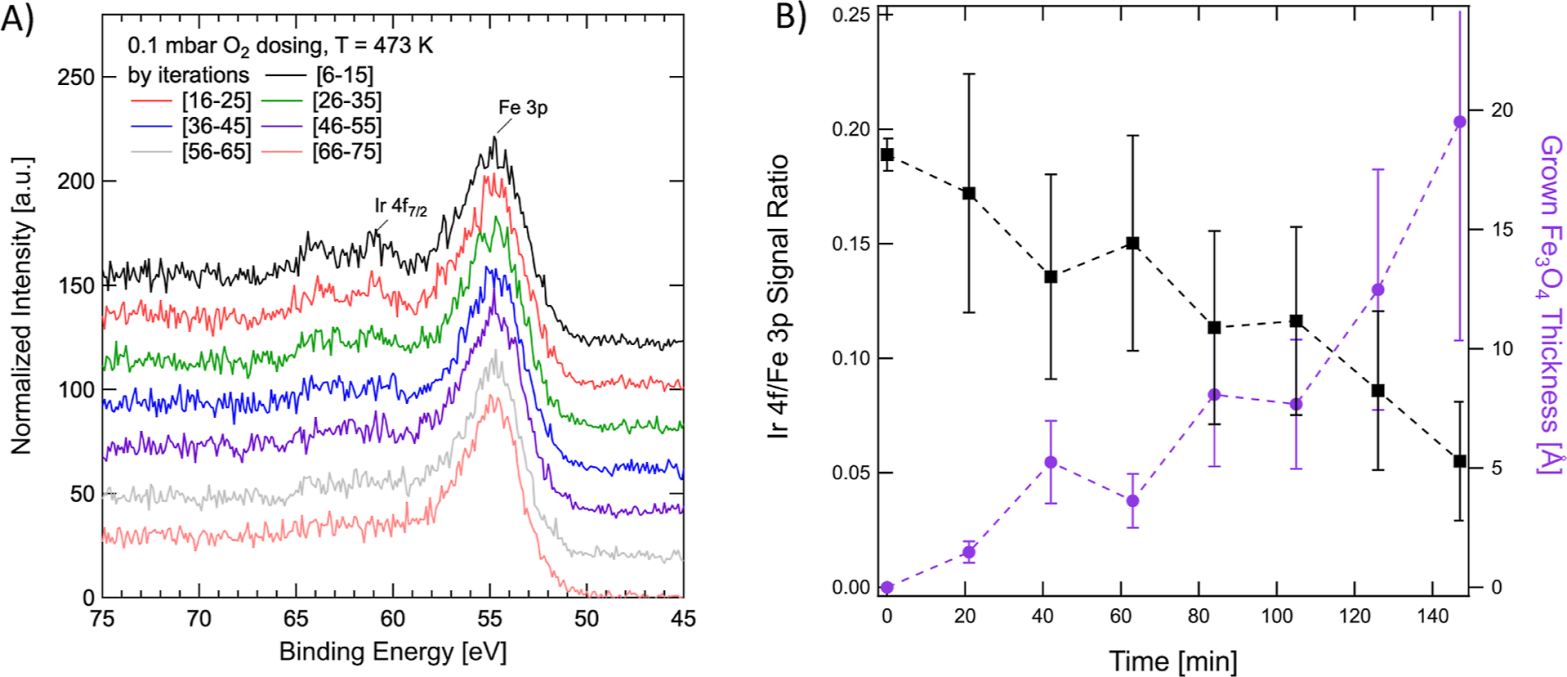
(A) Encapsulation of Ir_1_ in 0.1 mbar O_2_, followed through a time-lapsed XPS measurement lasting approximately 160 min in total. (B) Calculated ratio of Ir 4f/Fe 3p signal as a function of time, and estimated effective thickness of Fe_3_O_4_ grown to induce the signal reduction.

To identify the reason for this change, we investigated how annealing to 473 K affected the surface while increasing the partial gas pressures. Upon reaching 0.1 mbar O_2_ at 473 K, the intensity of the Ir 4f peak began decreasing over time. We attribute this phenomenon to the encapsulation of Ir_1_ in the Fe_3_O_4_ surface at sufficiently high temperature and O_2_ partial pressure. The IMFP for electrons originating from the Ir 4f through the Fe_3_O_4_ is 1.8 nm. With the probing depth being 3× the value of the IMFP, the disappearance of the Ir 4f signal corresponds to a Fe_3_O_4_ overlayer with a thickness of at least 5.4 nm. While Ir_1_ is stable at this temperature in the two-fold coordinated adsorption site in vacuum, Fe cations from the near-surface region still possess sufficient mobility to diffuse to the surface.^51^ Excess Fe cations that have diffused to the surface then react with dissociated O, which results in the growth of additional iron oxide layers.^29^Figure 6 follows this progress over the course of several measurement iterations. From the attenuation of the original Ir 4f intensity, it is possible to estimate an average thickness of oxide grown over the Ir_1_ adatoms. It should be noted, however, that once a first layer of oxide is grown over Ir_1_, the Ir atom may start diffusing into subsurface layers. We do not observe significant variations in the Fe 3p or O 1s spectra to identify different oxidation states for Fe.

**Figure 7:**
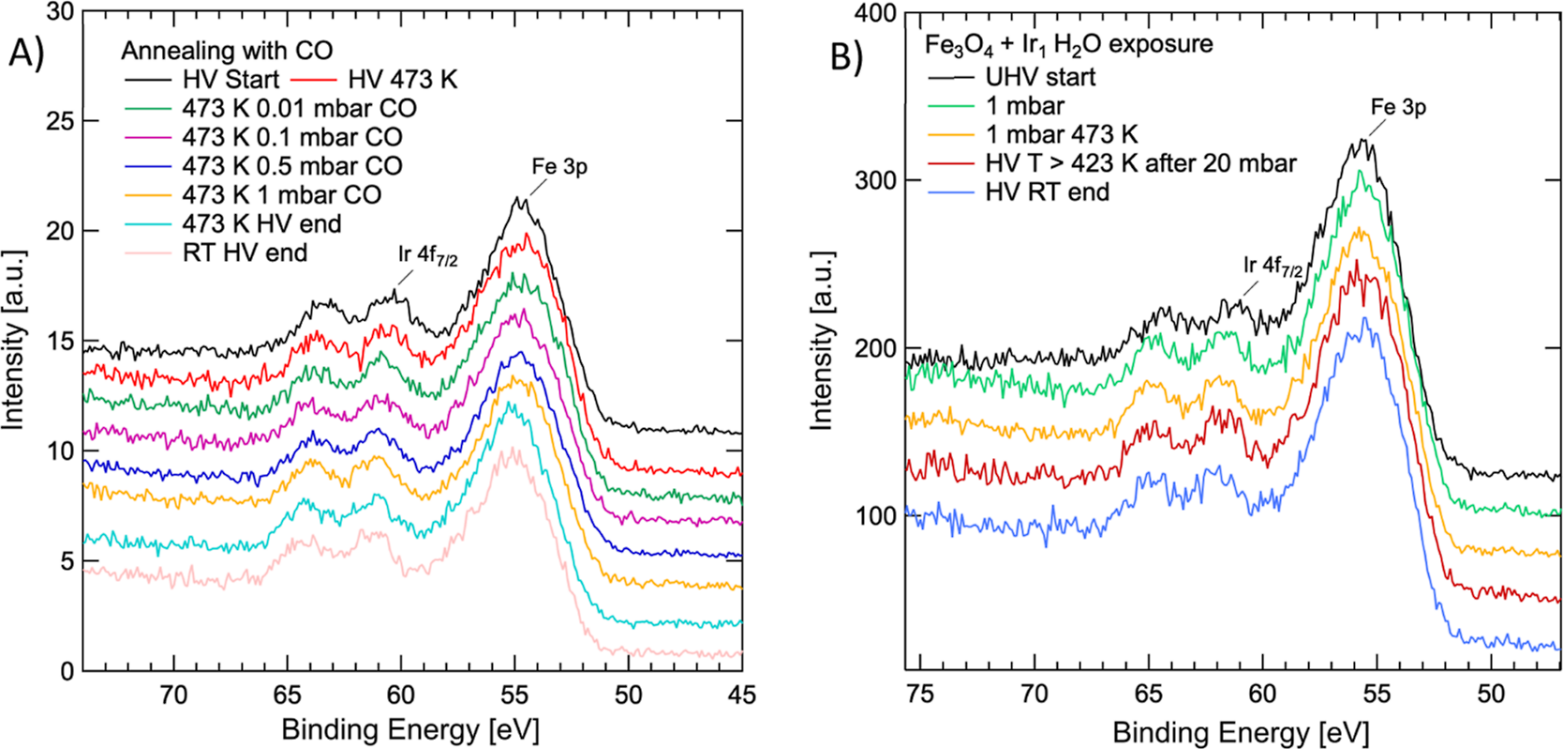
APXPS spectra of the Fe 3p/Ir 4f region under exposure to up to 1 mbar CO (A) and 20 mbar H_2_O (B). In neither case, Ir_1_ encapsulation is observed at a temperature of 473 K.

A similar experiment was performed in CO and H_2_O. The reducing nature of CO should not induce magnetite growth. Surface hydroxyls from water dissociation may either inhibit encapsulation by stabilizing the surface or provide the oxygen atoms required. Figure 7 shows APXPS spectra taken at a sample temperature of 473 K for both gasses at different pressures. In all cases, the intensity of the Ir 4f peaks is unaffected and we thus conclude that encapsulation in the examined temperature range may take place only under strongly oxidizing conditions. Interestingly, for water exposure at 1 mbar, there is no difference in the Ir 4f peak position or the amount of C1s for the data acquired at room temperature (green in Figure 7b) and at 473 K (yellow in Figure 7b), and the only difference we can observe is the absence of the adsorbed water at ∼535 eV in the O 1s spectra due to the decrease of the relative humidity on the surface at 473 K (see the Data Availability section). In the absence of sufficient partial oxygen pressure and thermal energy, Ir_1_ remains stable on the Fe_3_O_4_(001) surface. These results indicate that the bond between Ir_1_ and CO is too strong for the Ir_1_–Fe_3_O_4_(001) model catalyst, and other adatoms with a weaker CO bond, such as Pt or Rh, might be more suitable for CO oxidation.^28^

## 4 Conclusions

We investigated Ir_1_ single atoms on a Fe_3_O_4_(001)–(√2 × √2)R45° surface both in UHV and upon exposure to different common chemical processing gases (O_2_, CO, H_2_O, H_2_, N_2_, and Ar). Shifts in Ir 4f BE correlate with adventitious carbon deposition occurring during the measurements, indicating coordination of Ir_1_ to these C atoms, likely facilitated by surface hydroxyls.^45^ Ir_1_ coordination with adventitious C can occur together with CO adsorption and results in a different Ir_1_ configuration as indicated by a higher core electron BE. No clear evidence of gas molecule-Ir_1_ bonding can be observed by APXPS, due to the low Ir_1_ coverage and signal-to-noise ratio. Ir_1_ is observed to remain stable on the support at room temperature when exposed to up to 1 mbar of O_2_, CO, H_2_, N_2_, and Ar, and up to 20 mbar of water vapor. Ir_1_ adatoms are stable even in the presence of a large amount of adventitious carbon, which further emphasizes their stability outside the UHV conditions, and such a system could be potentially used as a catalyst under realistic reactor conditions. At 473 K, encapsulation of Ir_1_ occurs in a pressure of 0.1 mbar O_2_ or higher, as Fe cations from the near-surface can diffuse to the surface layer and new oxide layers are grown. This encapsulation does not occur in non-oxidizing environments or in pure water vapor. No significant reduction or instability of Ir_1_ is observed in reducing environments such as 1 mbar H_2_ (RT) or 1 mbar CO (up to 473 K). While in situ catalytical oxidation of CO over Ir_1_ could not be observed, these results offer an initial understanding of single-atom stability at near-ambient pressures.

## Supporting Information Available

Equation used to determine the coverage of adventitious carbon; LEED of a clean surface; additional APXPS spectra; Ir 4f BE shift as a function of C thickness; Ir 4f BE shift vs the HV annealing temperature; and bar graph including the coverage of C and N during increasing pressure of N_2_ gas (PDF)

## Notes

The data presented in this paper are available via DOI:10.5281/zenodo.7257864.
