# Supplementary material for: Stability of Iridium Single Atoms on Fe3O4(001) in the mbar Pressure Range
Source: J Phys Chem C Nanomater Interfaces. 2023 Sep 14;127(38):19097–106. doi: 10.1021/acs.jpcc.3c03097 (PMC10544020; doi:10.1021/acs.jpcc.3c03097)
Supplement: Supplementary file 1 — jp3c03097_si_001.pdf [file jp3c03097_si_001.pdf]

Supporting Information

Stability of Iridium Single Atoms on $Fe_3O_4(001)$ in the mbar Pressure Range

Nicolo Comini[1,2], J. Trey Diulus[1,2], Gareth S. Parkinson[3], Jürg Osterwalder[1],*

Zbynek Novotny[1,2,4]*

1 – Physik-Institut, Universität Zürich, CH-8057 Zürich, Switzerland

2 – Swiss Light Source, Paul Scherrer Institut, CH-5232 Villigen-PSI, Switzerland

3 – Institute of Applied Physics, Technical University of Vienna, A-1040 Vienna, Austria

4 – EMPA, Laboratory for Joining Technologies and Corrosion, Swiss Federal Laboratories for Materials, CH-8600 Dübendorf, Switzerland

*Corresponding author. Email: zbynek.novotny@empa.ch, parkinson@iap.tuwien.ac.at

Equation S1: the following equation has been utilized to numerically determine the average thickness "d" of deposited carbon during an XPS measurement. The subscripts C and O indicate the deposited carbon and substrate oxygen, respectively:

$$I_C / I_O = [(\rho_C\ \sigma_C\ T\ \lambda_{C\text{-}C}) / (\rho_O\ \sigma_O\ \lambda_{O\text{-}Fe})] \times [(1 - e^{-d/(\lambda_{C\text{-}C} \cos\theta)}) / (e^{-d/(\lambda_{O\text{-}C} \cos\theta)})] \times e^{(-1/\lambda_{C\text{-}gas})}/e^{(-1/\lambda_{O\text{-}gas})}, \quad \text{Eq.S1}$$

where I represents the peak intensity, ρ_C the atomic density of carbon atoms in graphite and ρ_O of oxygen atoms in Fe_3O_4, T is the analyzer transmission function ratio (approximately equal to 1.2), $\lambda_{O\text{-}Fe}$ is the inelastic mean free path (IMFP) of O 1s photoelectrons in the $Fe_3O_4(001)$ substrate, $\lambda_{C\text{-}C}$ and $\lambda_{O\text{-}C}$ are the IMFPs respectively of C 1s and O 1s photoelectrons in graphitic carbon, $\lambda_{C\text{-}gas}$ and $\lambda_{O\text{-}gas}$ the IMFPs of the same photoelectrons in the gas phase, the σ_C and σ_O the differential cross-sections, and θ the polar emission angle. The IMFP of electrons in gas phase (units of mm), relevant only at pressures of 1 mbar, has been determined by bulk signal attenuation.

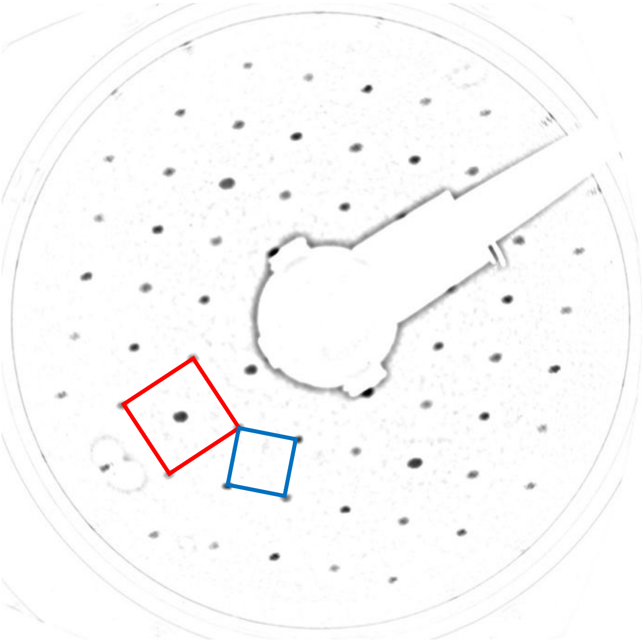

Figure S1. LEED pattern of a freshly prepared Fe_3O_4(001) presenting the characteristic (√2×√2)R45° reconstruction acquired with an incident electron energy of 100 eV. The unreconstructed surface unit cell (UC) is shown in red, and the reconstructed one in blue. The evaporation of < 1 Ir_1/UC does not modify the LEED pattern in any perceptible way. The image was processed to remove the radial distortion associated with the planar channel plate detector and turned b/w for improved visibility.

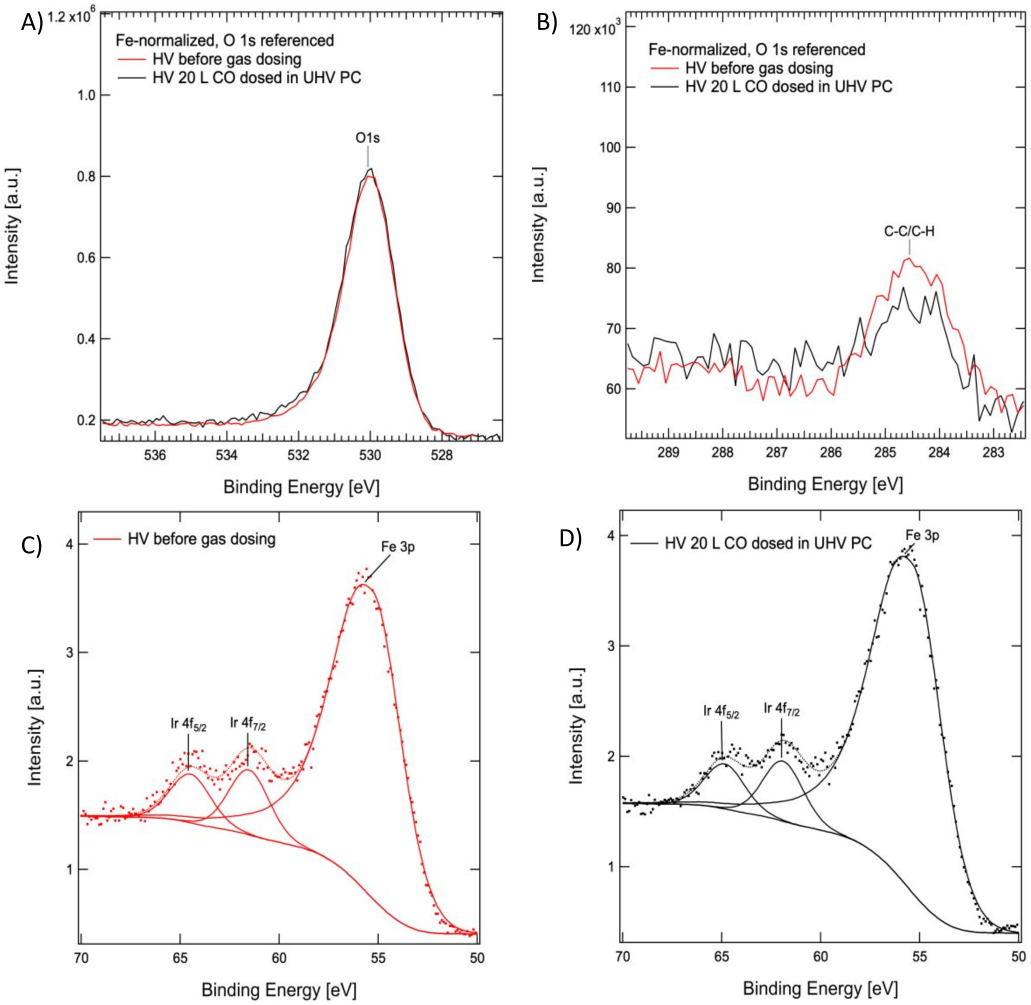

Figure S2. HV XPS spectra covering the O 1s region (A), and C 1s region (B) complementary to the data shown in Figure. 1. Panels (C) and (D) contain fitted spectra of the Ir 4f and Fe 3p region shown in Figure 1. Spectra contain Shirley background, measured points are plotted as large dots, and the peak envelope is shown as a dotted line. The Fe 3p peak is fitted with a GL(30)T(1.5) function with a FWHM of 3.4±0.1 eV. Ir 4f was fitted with a pair of GL(30) functions with constrained area ratio, spin-orbit splitting and having the same FWHM for both 7/2 and 5/2 component (in both cases being 2.4±0.1 eV). For HV data before gas dosing (C), the Ir $4f_{7/2}$ = 61.53 eV, while for data after CO dosing (D), Ir $4f_{4/2}$ = 61.91 eV.

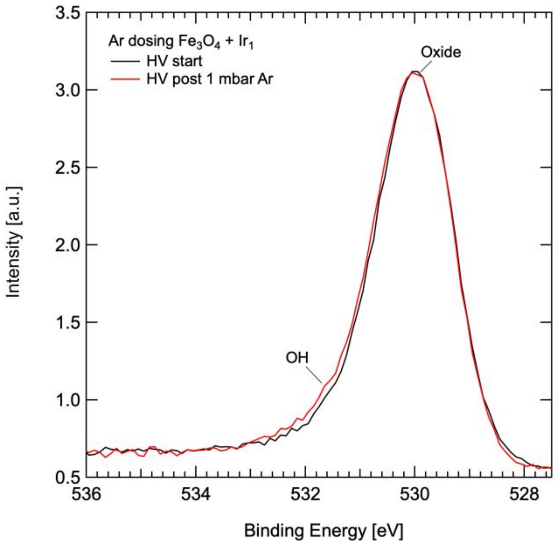

Figure S3. XPS of the O 1s region measured in HV before (black) and after (red) exposing the surface to 1 mbar of Ar. Both spectra are intensity normalized.

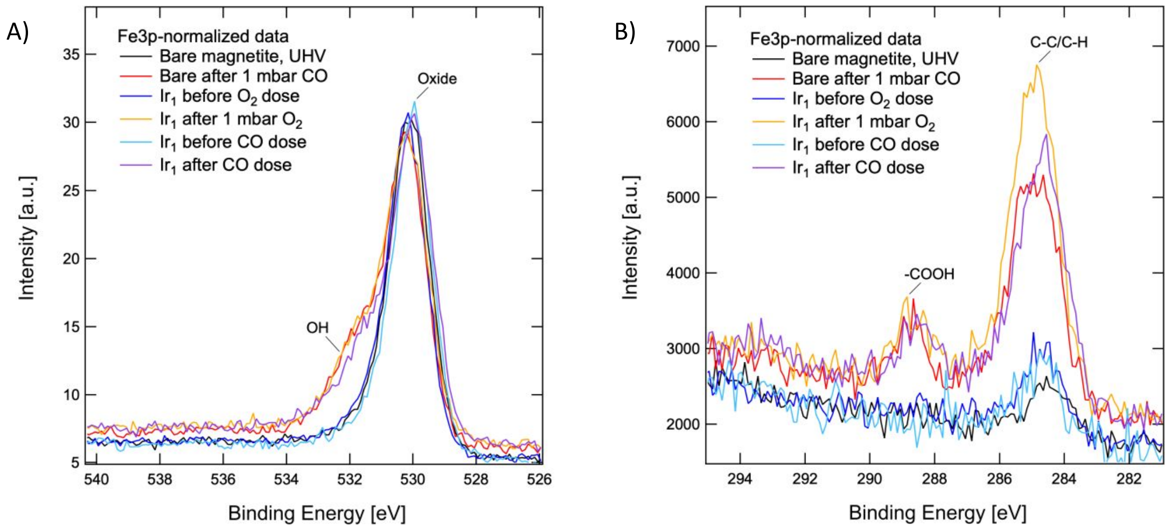

Figure S4. XPS spectra acquired in HV in the O 1s (A) and C 1s (B) regions before and after CO and O_2 exposure. No difference is readily observable between spectra, except for slightly different levels of adventitious carbon contamination, even compared to an experiment where no Ir was evaporated on the surface (red and black). Exposure to O_2 on a bare surface showed an identical peak envelope for Fe 3p and O 1s as when Ir_1 was present (data not shown). O-

containing carbon species adsorb on the surface at higher pressures, which correlate to the increase of a higher-BE shoulder in the O 1s region.

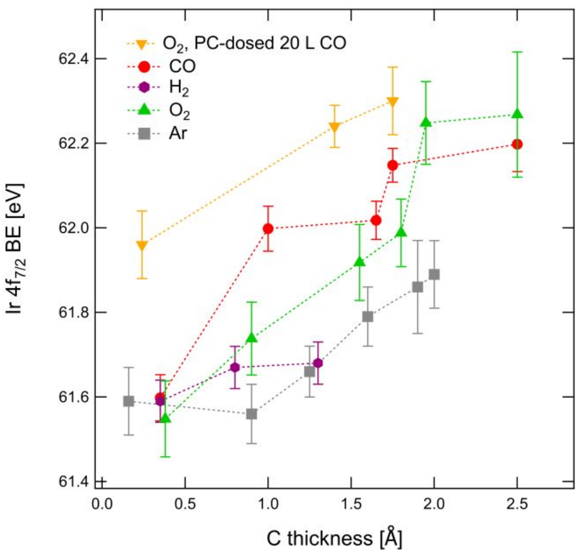

Figure S5. Ir $4f_{7/2}$ BE as a function of the surface carbon coverage for a selection of test gas exposures: Ar (gray), CO (red), H_2 (purple), O_2 (green) and O_2 after a 20 L CO dose performed in UHV in the sample preparation chamber (yellow). Carbon is assumed to take a graphite-like structure for determining its thickness. Data suggests a linear relationship between Ir BE and carbon coverage, up to a full layer. Note that *in-situ* exposure to CO even at the lowest possible experimental pressures (10^{-3} mbar, corresponding to the second point in the graph, due to the opening limit of a flowmeter) results in a rapid change to higher BE similar to what is observed after a limited dose in UHV. The first and last data points in each curve represent measurements in HV, while the second-last data point represents the exposure to 1 mbar. The absence of reversibility of the Ir 4f BE shift between the measurement at 1 mbar and subsequent UHV measurement indicates that the changes on the Ir_1 are irreversible, and therefore could be studied *ex-situ* using a standard UHV system equipped with a high-pressure cell.

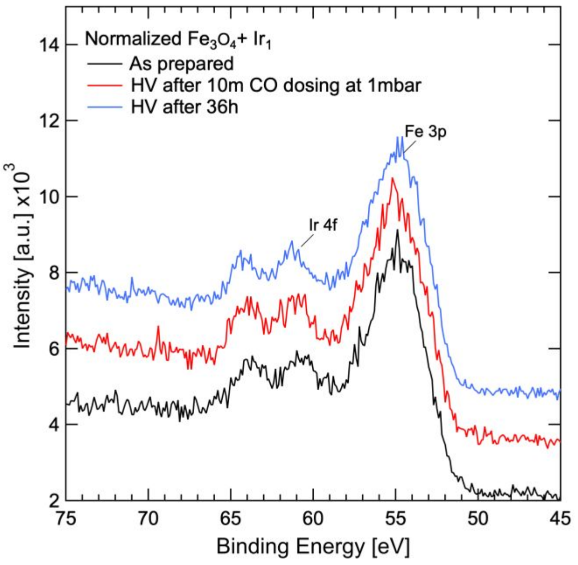

Figure S6. XPS spectra of the Ir 4f and Fe 3p region acquired in HV immediately after preparation (black), after a brief exposure to 1 mbar CO (red), and after storage in UHV for 36 h (blue). Aside from the initial Ir 4f BE shift occurring upon exposure to CO, no significant change takes place over time in vacuum.

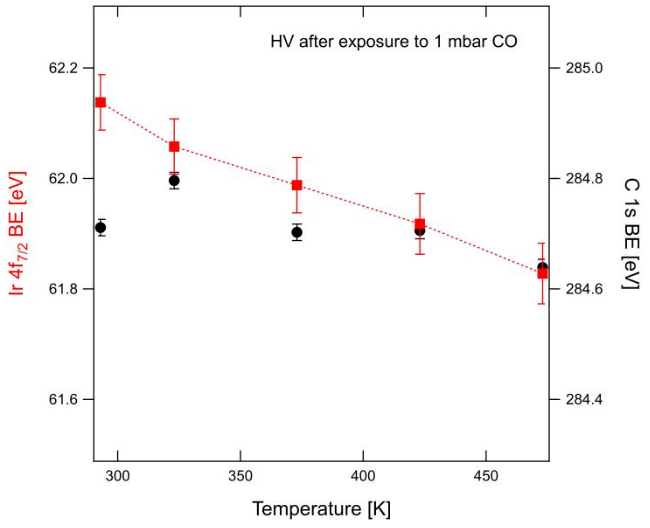

Figure S7. Ir $4f_{7/2}$ BE (red) and C 1s BE (black) during annealing in HV (~10^{-6} – 10^{-7} mbar), after exposure to 1 mbar CO. The Ir peak energy position is slightly reduced as temperature increases. As the temperature is insufficient to desorb CO from Ir_1,[1] this is likely due to

restructuring of the adventitious carbon and carbon-containing species distributed on the surface near the adatom.

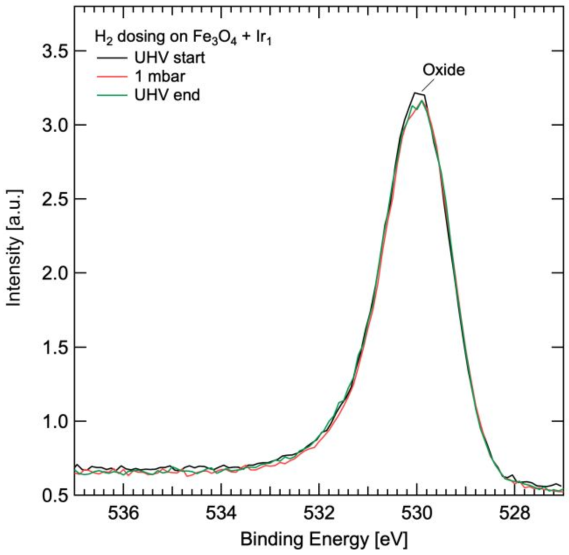

Figure S8. XPS of the O 1s region measured in HV before (black), and after (green) exposing the surface to 1 mbar of H_2. The spectrum acquired during 1 mbar H_2 exposure is shown in red. All spectra are intensity normalized.

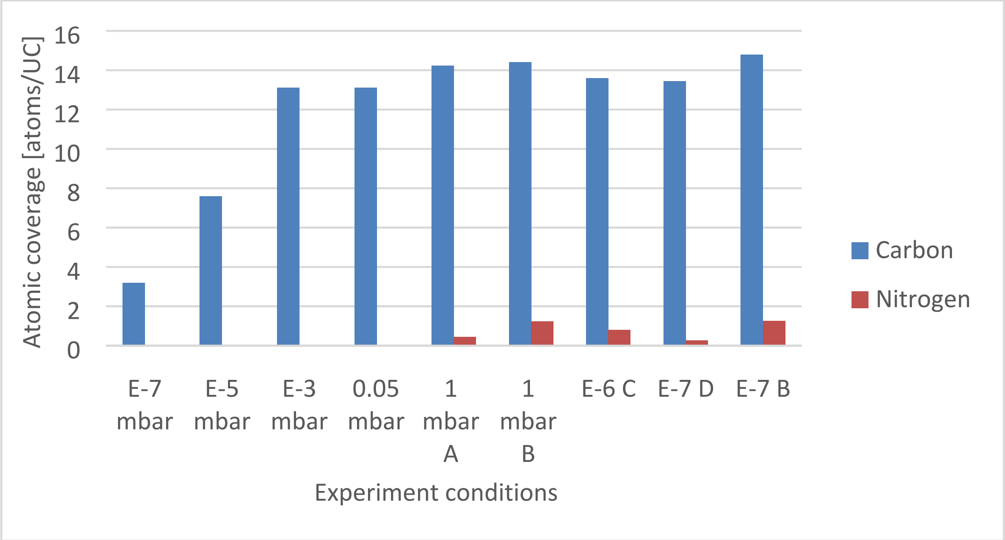

Figure S9. Histogram reporting carbon and nitrogen coverage during exposure of bare $Fe_3O_4(001)$ to N_2. While carbon increases with the pressure, no significant changes occur after reaching ~1 mbar of N_2. Different spots on the sample surface at a distance larger than the beam size (here labeled as A, B, C and D) show a different surface concentration of

adsorbed N. Adsorption may be hindered by the presence of carbon on the surface, but not as a sole factor.

REFERENCES

(1) Jakub, Z.; Hulva, J.; Meier, M.; Bliem, R.; Kraushofer, F.; Setvin, M.; Schmid, M.; Diebold, U.; Franchini, C.; Parkinson, G. S. Local structure and coordination define adsorption in a model Ir_1/Fe_3O_4 single-atom catalyst. *Angewandte Chemie* **2019**, *131* (39), 14099-14106. DOI: 10.1002/ange.201907536.
